# Breeding Thin-Billed Prions Use Marine Habitats Ranging from Inshore to Distant Antarctic Waters

**DOI:** 10.3390/ani12223131

**Published:** 2022-11-13

**Authors:** Petra Quillfeldt, Andreas Bange, Aude Boutet, Rachael A. Orben, Alastair M. M. Baylis

**Affiliations:** 1 Department of Animal Ecology & Systematics, Justus Liebig University Giessen, Heinrich-Buff-Ring 26, 35392 Giessen, Germany; 2 Department of Fisheries, Wildlife, and Conservation Sciences, Hatfield Marine Science Center, Oregon State University, Newport, OR 97365, USA; 3 South Atlantic Environmental Research Institute, Stanley FIQQ 1ZZ, Falkland Islands

**Keywords:** seabird tracking, GPS, foraging areas, marine habitats

## Abstract

**Simple Summary:**

Thin-billed Prions are small seabirds with large foraging ranges. Thin-billed prions from the Falkland Islands were tracked with Global Positioning System (GPS) dataloggers during the breeding season. During incubation trips, Thin-billed Prions travelled distances of approx. 2000 km, and foraged on the Patagonian Shelf or in Polar Front waters. During chick-rearing, Thin-billed Prions undertook trips of variable duration (one to 11 days), and foraged more locally, including in inshore waters. Birds from two colonies used spatially segregated foraging areas.

**Abstract:**

Pelagic seabirds cover large distances efficiently and thus may reach a variety of marine habitats during breeding. Previous studies using stable isotope data and geolocators suggested that Thin-billed Prions breeding in the Falkland Islands in the Southwest Atlantic may forage in temperate waters over the Patagonian Shelf or cross the Drake Passage to forage in Antarctic waters south of the Polar Front. We deployed miniature GPS dataloggers to track Thin-billed prions in the Falkland Islands during incubation (3 seasons) and chick-rearing (2 seasons). Thin-billed Prions had a wide distribution during incubation, covering latitudes between 43 and 60° S, with trip lengths of ca. 2000 km over seven days, on average. Thin-billed Prions from two nearby sites (60 km apart) were spatially segregated in their incubation trips, with New Island Thin-billed Prions foraging over the Patagonian Shelf, compared to Thin-billed Prions from Bird Island, that foraged in the region of the Polar Front. During chick-rearing, Thin-billed Prions from New Island undertook both long trips to the Patagonian Shelf and south of the Polar Front (30% of trips were 5–11 days), and short trips (70% of trips were 1–4 days) when they foraged more locally, including in inshore waters around the Falkland Islands. Females carried out more trips to distant sites. Thus, Thin-billed showed a high flexibility in foraging areas, habitats and foraging trip durations, which enable them to benefit from both, temperate and Antarctic environments.

## 1. Introduction

Southern Ocean seabirds experience a discrepancy between a vast, albeit highly heterogeneous marine environment and the limited availability of suitable breeding habitat, which is often in the form of remote and uninhabited islands. Islands can therefore, host large populations of seabirds. Where neighbouring colonies are within the foraging range of one another, inter-colony segregation in foraging areas may be apparent (e.g., [1,2]). While limited in area relative to the pelagic habitats of the Southern Ocean, the Patagonia Shelf, the Kerguelen-Heard Plateau, and the Campbell Plateau all support large and diverse seabird populations. Here, seabird foraging distributions are concentrated in zones of enhanced production such as tidal fronts and shelf slopes. In the pelagic Southern Ocean, several seabird and prey species concentrate in predictable frontal zones such as the Polar Front (e.g., cephalopods [3], King Penguins *Aptenodytes patagonicus* [4,5], seabird assemblages [6], Common Diving Petrels *Pelecanoides urinatrix* [7]). However, pelagic frontal zones are often located far from breeding colonies, and may not be easily accessible for central place foraging colonial breeding seabirds.

During the breeding period, the major constraint on breeding adult seabirds is the requirement to return to the colony to incubate the egg, or to feed a nutritionally dependent chick. However, the time constraints and thus optimal foraging strategies, change depending on breeding stage. For example, during incubation, one adult is free from parental duties for several days while its partner incubates the egg. During chick-rearing, both parents need to collect prey for themselves and to provision their offspring. Hence, the distance between the breeding colony and highly productive zones could reduce the offspring provisioning rate to unsustainable levels and thus many pelagic seabirds have developed a dual foraging strategy during chick-rearing [8] consisting of long and short trips. This enables them to exploit both foraging grounds close to the breeding site and productive distant areas such as the Polar Frontal Zone. Self-feeding requirements and resultant trip durations are also expected to differ by sex as females initially invest more energy in egg laying, while males may spend more time guarding nest sites. However, dual foraging is expected to manifest when the frequency of feeding small chicks necessitates foraging in local areas that may not have resources to support both chick feeding and self-maintenance.

We previously investigated the pre-laying and early incubation time of Thin-billed Prions *Pachyptila belcheri* using light geolocators [9,10], that have a relatively low spatial resolution. According to this study, incubation trips of Thin-billed Prions from New Island, Falkland Islands, lasted 7.3 ± 1.7 days, with a total distance covered of 2737 (±833) km. During incubation trips, Thin-billed Prions from New Island reached estimated maximum distances from the colony of 883 (±399) km, with a mean daily travel speed of 375 (±82) km/day and mostly foraged over the Patagonian Shelf during incubation. Very few individuals crossed the Drake Passage to forage in Antarctic waters south of the Polar Front. However, a dual foraging strategy has been observed in chick-rearing Thin-billed Prions at Kerguelen [8], consisting of short trips ranging from 1–3 days and long trips of 4–9 days. Similar trip lengths during chick-rearing were registered previously using radiotracking in the New Island colony [11]. Thus, we anticipate that some individuals may use a dual foraging strategy during early chick-rearing.

In the present study, we used Global Positioning System (GPS) data loggers to determine the foraging areas of Thin-billed Prions during incubation and chick-rearing. In particular, we aimed to test the following hypotheses: (1) Thin-billed Prions use foraging grounds close to the breeding site and productive distant areas such as the Polar Frontal Zone, (2) A dual foraging strategy is observed during chick-rearing, but not during incubation, (3) Thin-billed Prions have sex-specific foraging strategies and (4) Thin-billed Prions from different colonies segregate spatially.

## 2. Materials and Methods

### 2.1. Study Species and Sites

The study was carried out at the New Island National Nature Reserve, Falkland Islands (51°43′ S, 61°17′ W) during the breeding seasons 2017–2018, 2018–2019, 2019–2020 and 2021–2022, and at Bird Island National Nature Reserve, Falkland Islands (51°43′ S, 61°17′ W) during the breeding season 2021–2022. Thin-billed Prions are small petrels, breeding mainly on the Falkland and Kerguelen Islands. New Island, in the Falkland Islands, is the largest known breeding site for Thin-billed Prions with an estimated 2 million breeding pairs [12]. Bird Island is estimated to hold ~600,000 breeding pairs [13]. The life cycle and basic biology of Thin-billed Prions were described by Strange [14]. They show the typical procellariiform pattern of a single-egg clutch and slow chick development. More recently, the movement ecology of Thin-billed Prions was analysed in the Falkland and Kerguelen Islands using geolocation loggers. The non-breeding distribution includes a migration to moulting sites in the Atlantic sector of the Southern Ocean and while Kerguelen birds remain there, most birds from the Falkland Islands spend the second part of the non-breeding season over the Patagonian Shelf [15,16,17], which is also used during the early breeding season [9,10].

The diet of Thin-billed Prions from the Falkland Islands and Kerguelen during chick-rearing consists predominantly of pelagic crustaceans (in order of importance: *Themisto gaudichaudii*, *Euphausia vallentini*, *Munida gregaria*, *Calanus simillimus*, larvae of Cirripedia) and minor amounts of cephalopod tissue [11,18]. During incubation, the cephalopod *Gonatus antarcticus* is the dominant diet component in the Falkland Islands [11].

### 2.2. Field Methods

To investigate spatial movements, we attached tail-mounted miniaturized GPS dataloggers (Pathtrack Otley, UK or Lotek, Wareham, UK) to breeding adult Thin-billed Prions. The loggers (Pathtrack nanoFix^®^ GEO-Mini 0.95 g and <2 g, and Lotek PinPoint 10 < 1.4 g) were attached to the four central tail feathers using Tesa tape (Tesa SE, Norderstedt, Germany) (for sample sizes, see Table 1). Nests were selected according to accessibility, and at New Island, the presence of individuals known from previous years, to maximize the chances of recapture. The recapture rate of birds with tail-mounted GPS dataloggers (after 9 to 20 days) varied between 81% and 100% (Table 1), and failures to recover were connected to nest abandonment. Initially, we also tried harness attachments (breeding seasons 2017–2018 and 2018–2019), but 9 of 10 chick-rearing adults abandoned (Table 1) and thus harness attachments are strongly discouraged in the species. Of the recovered devices, data were successfully obtained from 45 PathTrack nanoFix deployments (11 on Bird Island and 34 on New Island) and 25 PinPoint GPS (on New Island). Complete tracks were obtained more successfully from PathTrack nanoFix deployments (N = 40 or 89%), than from PinPoint GPS (N = 9 or 36%).

Birds from New Island were sexed from blood or feather samples (N = 20 females, 16 males), based on differences in length between introns in the CHD-Z and CHD-W genes and we used the primers 2550F/2718R [19].

### 2.3. Data Analyses

Data analyses were carried out in R version 4.1.0 [20], and visualized in R or ArcGIS 10.2.2 [21]. The raw data obtained were divided into trips (1–8 per bird) and checked for completeness (from nest departure to the return to the colony). Most tags recorded GPS positions every 3 h, or were interpolated to a common step length of 3 h. Complete trips (N = 86) were passed through behaviour classification using hidden Markov models in the R package moveHMM [22]. Two behaviour states (travelling vs. foraging) were defined based on step lengths and turning angles. The foraging locations were used in kernel analyses, using the R package adehabitatHR [23], with the settings h = “href”, kern = “bivnorm”. Trip lengths and maximum distances to the colony were calculated in ArcGIS, after projecting to South Pole Azimuthal Equidistant projection. Overlap between core foraging areas (50% kernels) or home range areas (95% kernels) were calculated in ArcGIS, after projecting to South Pole Lambert Azimuthal Equal Area projection.

**Figure 1:**
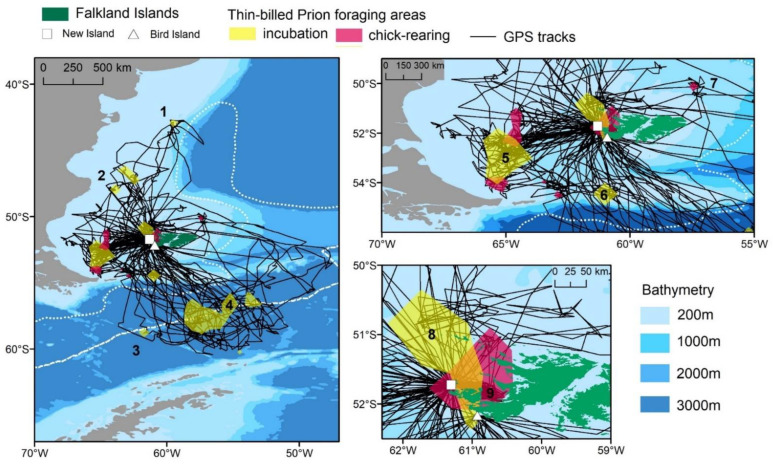
Overview map of tracks and main foraging areas (obtained by kernel analysis) of Thin-billed Prions from two colonies in the Falkland Islands.

The foraging areas were classified for each trip according to the furthest foraging area reached (Figure 1, Table 2), the direction relative to the colony (e.g., South, North-west, etc.) and the habitat classes “coastal” (i.e., in West Falkland waters), “Patagonian Shelf” (i.e., within the <200 m depth area), “Shelf break” (i.e., the zone of rapid depth increase to >1000 m) and “Polar Frontal Zone (PFZ)” (Figure 1, Table 2).

Trip parameters were compared using Kruskal-Wallis ANOVA on Ranks, followed by Dunn tests, and between sexes, trip parameters were compared by *t*-test or Wilcoxon tests, depending on the tests of normality in data distribution.

## 3. Results

### 3.1. Distribution

**Figure 2:**
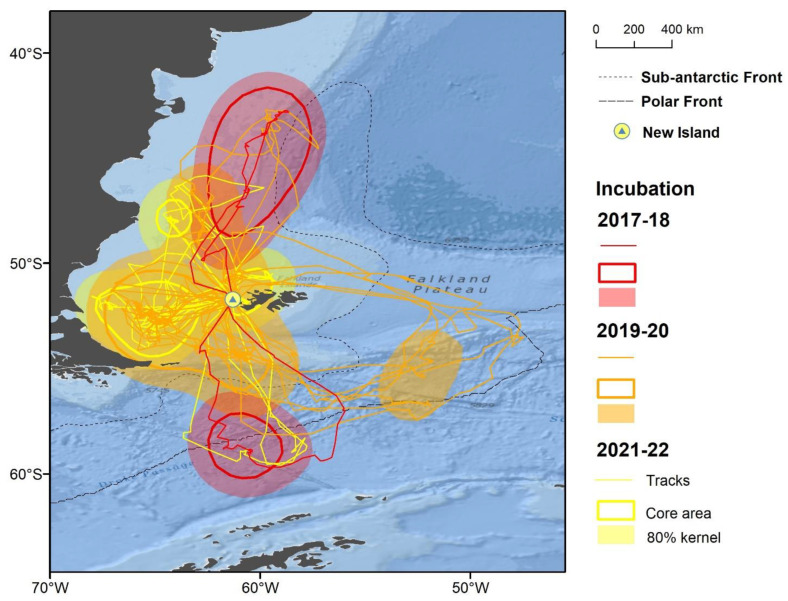
Tracks and main foraging areas (obtained by kernel analysis) of Thin-billed Prions from New Island during incubation, recorded in three breeding seasons. Area numbers refer to Table 3.

**Figure 3:**
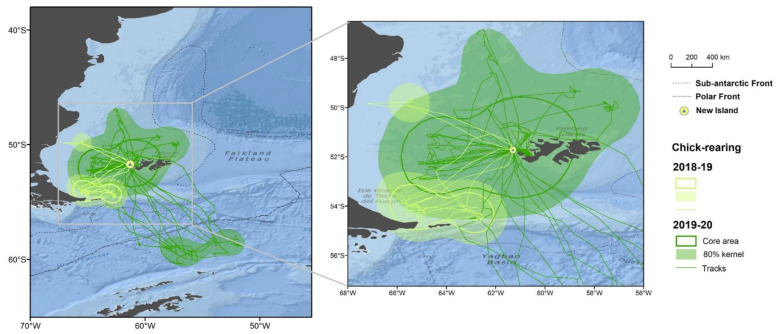
Tracks and main foraging areas (obtained by kernel analysis) of Thin-billed Prions from New Island during chick-rearing, recorded in two breeding seasons.

**Figure 4:**
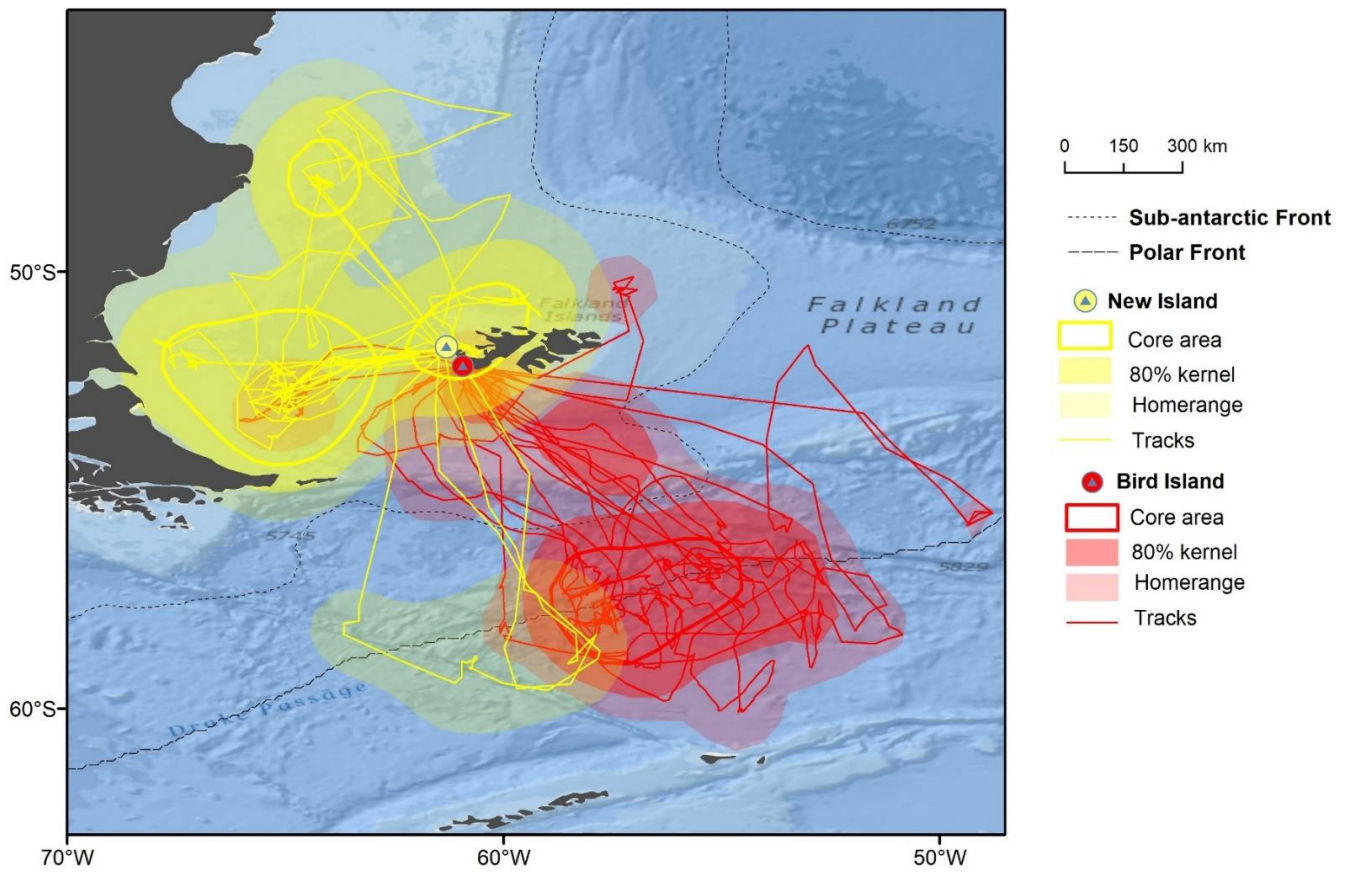
Tracks and main foraging areas (obtained by kernel analysis) of Thin-billed Prions from New Island and Bird Island during incubation in the season 2021–2022.

Thin-billed Prions had a wide distribution during the breeding season, covering latitudes between 42.7° S and 60.4° S, and longitudes between 68.0° W and 47.5° W (Figure 1). This entire area was used during incubation (Figure 2), while Thin-billed Prions foraged more locally during chick-rearing (Figure 3). The distribution of Thin-billed Prions from New Island and Bird Island during incubation 2021–22 were spatially segregated, with little overlap (Figure 4). There was no overlap between core areas, and only 13% overlap between the foraging home range areas of birds tracked from the two colonies.

### 3.2. Trip Parameters

Thin-billed prion incubation trips were longer in duration and total distance than chick-rearing trips (Table 2). During incubation, birds from both colonies carried out 91% long trips, compared to 30% during chick-rearing (Table 2). The long trips of Thin-billed Prions from Bird Island covered farther total distances, on average, than long trips of Thin-billed Prions from New Island (Dunn test: *p* = 0.046). Neither long nor short trip total distances differed between incubation and chick-rearing in Thin-billed Prions from New Island (Table 2).

The mean foraging ranges (i.e., maximum distance from the colony) differed between incubation and chick-rearing, but not between the sites (Table 2). However, long trips of Thin-billed Prions from Bird Island had, on average longer foraging ranges, when compared to the long trips of Thin-billed Prions from New Island (Dunn test: *p* = 0.024). The overall travel speed was higher for Thin-billed Prions from Bird Island (Table 2), as was the speed during long trips (Table 2). Moreover, Thin-billed Prions from New Island were faster during long chick-rearing trips than incubation trips (Table 2).

**Figure 5:**
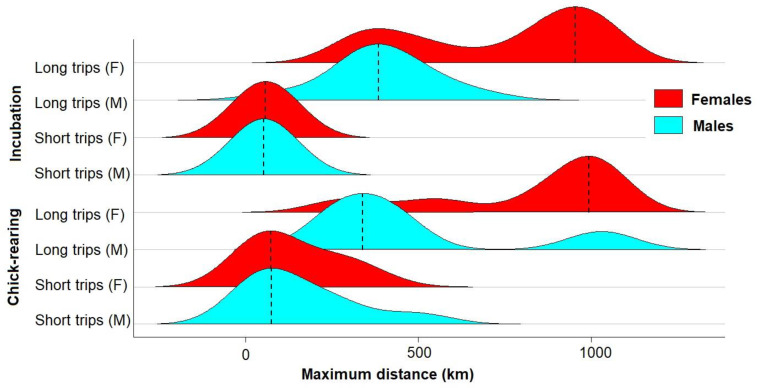
Ridgeline plot of sex differences in maximum distances of Thin-billed Prions from New Island, separately for the breeding phases and trip lengths (short = 1–4 days, long = 5–11 days). Dashed lines mark the mode.

In sexed birds from New Island, females performed longer trips on average than males during incubation (females: 2288 ± 899 km, males: 1517 ± 999 km, *t*-test: t = 2.1, df = 24.2, *p* = 0.045), and reached more distant sites from the colony (females: 694 ± 305 km, males: 341 ± 175 km, *t*-test: t = 3.9, df = 26.6, *p* < 0.001, Figure 5). This was achieved by higher speeds (females: 300.4 ± 91.4 km/day, males: 213.9 ± 101.6 km/day, *t*-test: t = 2.3, df = 24.3, *p* = 0.029), while trip durations did not differ (females: 7.6 ± 2.2 days, males: 6.7 ± 2.7 days, Wilcoxon test: W = 133.5, *p* = 0.338).

During chick-rearing, females from New Island carried out 28% long trips (7 of 25), compared to 62% in males (5 of 13, Chi-squared test: χ^2^ = 0.43, df = 1, *p* = 0.510). Short trips had similar ranges (Figure 5), but females tended to range further during long trips (females: 819 ± 266 km, males: 480 ± 283 km, *t*-test: t = 1.9, df = 8.2, *p* = 0.093, Figure 5).

### 3.3. Foraging Habitat Use

**Figure 6:**
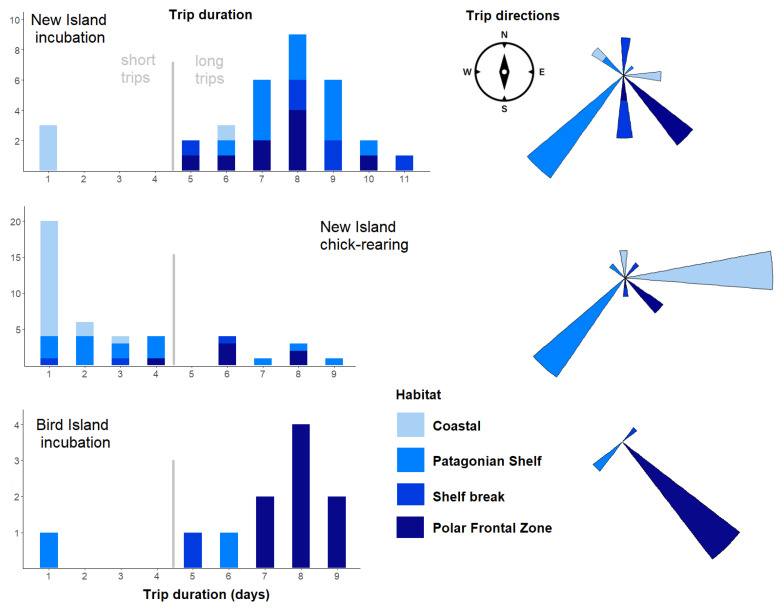
Habitat use of Thin-billed Prions from two colonies in the Falkland Islands, separately for the breeding stages. For a definition of the foraging areas, see Figure 1 and Table 2.

**Figure 7:**
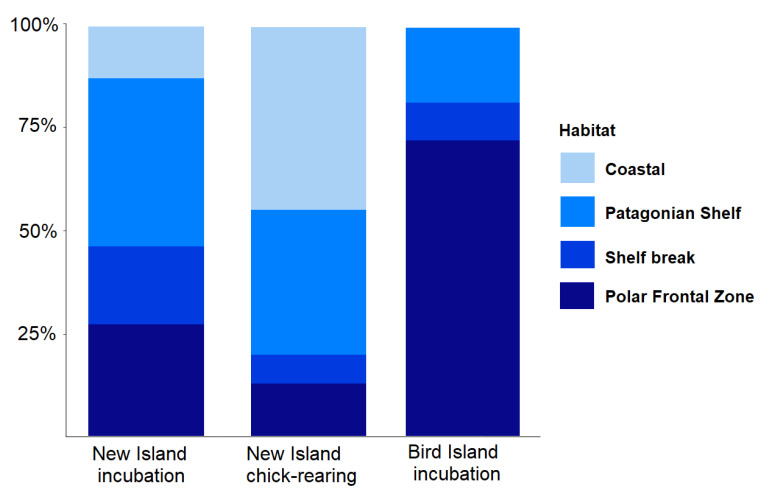
Habitat use of Thin-billed Prions from two colonies in the Falkland Islands, separately for the breeding stages, shown as circular histograms (polar plots) indicating the travel direction.

The habitat use differed between the colonies and stages (Table 3, Chi-squared test: χ^2^ = 25.1, df = 6, *p* < 0.001, Figure 6 and Figure 7). During incubation, Thin-billed Prions from New Island used the Patagonian Shelf habitat most frequently (41% of the trips), followed by the PFZ (28%). Thin-billed Prions from Bird Island, in contrast, predominantly (73%) used the PFZ, and less frequently used the Patagonian Shelf (18%). The Shelf break habitat was used during 19% and 9% of the incubation trips from New Island and Bird Island, respectively, and 7% of the chick-rearing trips from New Island. During chick-rearing, Thin-billed Prions from New Island used the coastal West Falkland waters most frequently (44% of the trips), followed by the Patagonian Shelf (35%).

## 4. Discussion

In the present study, we show that Thin-billed Prions from the Falkland Islands use foraging grounds close to the breeding site as well as the distant areas such as the PFZ. A dual foraging strategy was used predominantly during chick rearing, however sometimes short trips occurred during incubation, potentially a sign of egg neglect. Moreover, Thin-billed Prions from two nearby colonies segregated spatially during incubation with birds from the smaller colony at Bird Island more reliant on the PFZ. In the case of New Island, females used distant foraging sites more frequently than males during both incubation and chick rearing.

The different trip lengths allow Thin-billed Prions to exploit a range of productive habitats (Table 3). These included nearby coastal habitats around Queen Charlotte Bay and around the Jason Islands (depth 40–50 m, kernel area 9 in Figure 1), which were used by Thin-billed Prions from New Island during chick-rearing. These areas are thought to have a high abundance of amphipods. For example, Sei whales use these coastal waters as a seasonal feeding ground between January and May, preying on amphipods *Themisto gaudichaudii* and lobster krill *Munida gregaria* [24]. *Themisto gaudichaudii* is the most important prey of Thin-billed Prions, while *Munida gregaria* seems to be less preferred, and was found mainly in a year of low food availability [11].

Trips of intermediate length were directed towards feeding sites on the Patagonian Shelf (dept 100–200 m) in westerly and north-westerly direction (kernel areas 2, 5 and 8 in Figure 1). A productive marine system, with high mesozooplankton abundances (e.g., [25]), has been observed here, especially at 65–66° W longitudes (kernel area 5 in Figure 1). This is caused by an upwelling of Subantarctic Shelf Water (Sabatini et al. 2016) and high Chl *a* values associated with this mesoscale structure. Besides the smaller copepods that are only taken in poor conditions by Thin-billed Prions [11], this area harbours a high biomass of euphausiids (*Euphausia vallentini* and *E. lucens*) and amphipods *Themisto gaudichaudii* [25,26] and thus, the most important prey of Thin-billed Prions [11].

Further foraging areas were observed around the Shelf break and slope waters (i.e., water depths between 400 m and 800 m), in the north, south and east of the Falkland Islands (kernel areas 1, 6 and 7 in Figure 1). Shelf slope waters are also used regularly by Southern Rockhopper Penguins *Eudyptes chrysocome* during incubation [27], which have a similar diet to Thin-billed Prions, with a preference for squid *Gonatus antarcticus* in the incubation phase and crustaceans during chick-rearing [11]. *Gonatus antarcticus* is an abundant squid species that spawns in deep waters, while juveniles and subadults inhabit shelf and slope waters, as well as the Polar Frontal Zone (e.g., [28]).

Finally, the longest Thin-billed Prion trips were to the Polar Frontal Zone (kernel areas 3 and 4 in Figure 1), which is also a region of high zooplankton abundance (e.g., [29]). Similar to Thin-billed Prions, other Southern Ocean petrels exploit a wide variety of marine environments ranging from sub-tropical to Antarctic waters (e.g., White-chinned petrels *Procellaria aequinoctialis* from the Crozet Islands [30]). This has been mostly associated to a dual foraging strategy whereby adults exploit alternatively distant oceanic waters and closer shelf or slope waters. Such a dual foraging strategy, consisting of long and short trips, has been observed in many pelagic seabirds during chick-rearing [8]. In the present study, a dual foraging strategy was also observed most clearly during chick-rearing, but a smaller percentage of short trips were also registered during incubation. Dual foraging in Thin-billed Prions was previously proposed based on stable isotope values: carbon and nitrogen stable isotope values were intermediate between Antarctic and temperate waters, suggesting a mixed foraging strategy [31]. Moreover, tracking data from New Island using geolocators [9] showed most incubation trips over the Patagonian Shelf, but a few in southward direction across the Polar Front, in line with the present findings.

It has been proposed that long trips in a dual foraging strategy, are primarily for adult self-maintenance, while short trips are primarily for offspring provisioning. There is also some evidence that petrel parents show a coordinated performance within pairs and thus, avoid an overlap of long trips and minimize the risk of starvation of the chick (e.g., Wedge-tailed Shearwater *Puffinus pacificus* [32]). A follow-up study may thus attempt to track partners and weigh chicks simultaneously and confirm both the function of long and short trips in the context of provisioning and self-maintenance, and the coordinated performance of pair members in Thin-billed Prions. During incubation, the occurrence of short trips may be evidence of short-term egg neglect.

An alternative hypothesis is that foraging grounds close to the breeding site and more distant areas such as the Polar Frontal Zone are attended according to the climatic conditions, such as the prevailing winds. Wind speed and direction influence the energy expenditure during flight and search behaviour and may determine seabird movements, especially in pelagic petrels (e.g., [33,34]). At only ca. 130 g, Thin-billed Prions may strongly depend on favourable winds to forage efficiently. This would best be studied using a multi-year dataset covering a range of environmental conditions which affect the foraging rates in this species (e.g., [11]).

Another objective of the present study was to explore if Thin-billed Prions have sex-specific foraging strategies. The previous evidence was not conclusive. Sex differences were suggested by a stable isotope study [31], where males and females differed in carbon and nitrogen values during courtship and chick feeding, suggesting that on average, males foraged at a higher trophic level and further north than females. This was in line with the finding in the present study, namely that females originating from New Island reached longer mean distances from the colony than males (e.g., Figure 5). In a study using radio telemetry, males and females had similar foraging trip lengths, suggesting similar contributions to provisioning rates [35]. The two apparently contradictory studies can now be explained with the present results. Indeed, both studies are supported and explained by similar trip durations, but different foraging areas: Females moved faster than males (300 vs. 210 km/day) and thus, reached more distant sites (690 vs. 340 km), but the trip duration was similar. Sex-specific foraging strategies have also been found in other petrel species. In Barau’s Petrel *Pterodroma baraui*, sex differences were greater during pre-laying and incubation than during chick-rearing [36], and this was explained with the different parental roles: males take the first long incubation shift at the nest, while females need to restore their body condition after laying. This energy constraint may also shape the differences observed here, e.g., if females use the more distant sites to forage more efficiently and thus, replenish body reserves or specific nutrients. In this context, another interesting aspect would be possible diet differences between males and females in relation to differences in foraging, and further studies could use biomarkers or genetic diet determination, for example based on fecal samples (e.g., [37,38]).

The final objective of this study was to explore if Thin-billed Prions from different colonies segregated spatially. Data were available for the incubation period from New Island and nearby Bird Island. Despite the small distance of ca. 56 km between the sites, the two populations showed nearly complete spatial segregation, consistent with the “hinterland model” [39] and the “density-dependent hinterland (DDH) model” [40]. The “hinterland model” proposed that seabirds form neighbouring colonies typically occupy non-overlapping feeding zones, and that the colony size is a function of the size of these zones (e.g., [41]). In the DDH model, segregation of foraging areas of two neighbouring colonies will occur if competition is high due to large colony size and small distance from the colony. Thus, where colonies are close and when colonies are relatively large, segregation will be most complete, but the hinterlands may overlap in areas where inter-colony competition is low. Thus, overlap is expected in foraging areas of superabundant prey, or where colonies are small or distant from one another.

Distributions consistent with the DDH model have been found in many seabird species, such as cormorants [42], gannets [43] and penguins [44,45]. Hence, inter-colony segregation of seabird feeding areas are common, included species with both smaller (10–100 km) and larger (100–1000 km) foraging ranges [1]. The finding of inter-colony segregation of foraging areas during incubation between the two colonies studied, suggests that intraspecific competition for prey is sufficiently intense for the DHH mechanism to act. However, Thin-billed Prions from the smaller colony typically travelled farther. Because the strength of segregation may change during the course of the breeding season (e.g., [44,46]), an analysis of tracking data during the chick-rearing season would provide interesting additional information.

## 5. Conclusions

In summary, the current data show considerable flexibility in foraging strategies of a small petrel species with regard to trip length, distance and direction, but also suggest further studies are needed to fully understand the drivers and constraints that determine when a specific foraging strategy will be employed.

## Figures and Tables

**Table 1:** Deployment and recovery periods, sample sizes (N) and recovery rates of Global Positioning System (GPS)-tracked Thin-billed Prions.

Island	Period	Deployment Date	N	Recovery Rate	Recovery Date
New	Incubation 2017–2018	1/12–16/12	2	2 (100%) *	14/12–30/12
New	Chick rearing 2018–2019	25/1–28/1	10	1 (10%) *	17/2
New	Incubation 2019–2020	4/12–17/12	18	18 (100%)	13/12–27/12
New	Chick rearing 2019–2020	25/1–28/1	9	9 (100%)	4/2–12/2
New	Incubation 2021–2022	19/12–28/12	30	26 (87%)	27/12–7/1
Bird	Incubation 2021–2022	1/12–13/12	16	13 (81%)	12/12–22/12

* Harness attachment was used during 2017–18 and 2018–19.

**Table 2:** Trip parameters (mean ± SD) of incubation and chick-rearing trips of Thin-billed Prions. Trip parameters were compared using Kruskal-Wallis ANOVA on Ranks (degrees of freedom: df), followed by Dunn tests. Within lines, homogenous sub-groups are given in superscript letters. Range is the maximum distance from the colony. “Short trips” are trips from 1–4 days, while “long trips” lasted 5–11 days.

Parameter	Unit	New Island Incubation (N = 32)	New Island Chick-Rearing (N = 43)	Bird Island Incubation (N = 11)	Kruskal Wallis ANOVA (df = 2) or Fisher Test
Duration	days	7.1 ± 2.4 ^a^	2.8 ± 2.4 ^b^	6.8 ± 2.1 ^a^	χ^2^ = 34.1, *p* < 0.001
% Long trips		91%	30%	91%	Fisher test: *p* < 0.001
Trip length (all)	km	1966 ± 984 ^a^	846 ± 854 ^b^	2490 ± 977 ^a^	χ^2^ = 28.2, *p* < 0.001
Trip length (long)	km	2155 ± 830 ^a^	1895 ± 754 ^a^	2715 ± 700 ^b^	χ^2^ = 5.4, *p* = 0.070
Trip length (short)	km	142 ± 34	392 ± 342	235	χ^2^ = 1.5, *p* = 0.480
Range (all)	km	558 ± 308 ^a^	301 ± 310 ^b^	781 ± 305 ^a^	χ^2^ = 21.2, *p* < 0.001
Range (long)	km	610 ± 276 ^a^	655 ± 317 ^a^	846 ± 237 ^b^	χ^2^ = 5.4, *p* = 0.140
Range (short)	km	55 ± 7	147 ± 126	135	χ^2^ = 1.4, *p* = 0.500
Travel speed (all)	km/day	266 ± 104 ^a^	279 ± 149 ^a^	349 ± 69 ^b^	χ^2^ = 4.3, *p* = 0.110
Travel speed (long)	km/day	278 ± 101 ^a^	321 ± 121 ^b^	363 ± 54 ^b^	χ^2^ = 5.4, *p* = 0.070
Travel speed (short)	km/day	148 ± 39	261 ± 156	201	χ^2^ = 1.1, *p* = 0.580

**Table 3:** Foraging areas of GPS-tracked Thin-billed Prions. Area numbers refer to Figure 1. PFZ = Polar Frontal Zone.

Area	Habitat	Direction from Colony	New Island Incubation (N = 32)	New Island Chick-Rearing (N = 43)	Bird Island Incubation (N = 11)
1	Shelf break	North	3 (9%)	-	-
2	Patagonian Shelf	North-west	2 (6%)	2 (5%)	-
3	PFZ	South	2 (6%)	1 (2%)	-
4	PFZ	South-east	7 (22%)	5 (12%)	8 (73%)
5	Patagonian Shelf	South-west	11 (34%)	13 (30%)	2 (18%)
6	Shelf break	South	3 (9%)	1 (2%)	-
7	Shelf break	North-east	-	2 (5%)	1 (9%)
8	Patagonian Shelf	North	1 (3%)	-	-
9	Coastal (Queen Charlotte Bay, Jason Islands)	East, North-east	3 (9%)	19 (44%)	-

## Data Availability

The data are archived in Movebank.
